# Pain Expression Recognition Based on pLSA Model

**DOI:** 10.1155/2014/736106

**Published:** 2014-03-27

**Authors:** Shaoping Zhu

**Affiliations:** Department of Information Management, Hunan University of Finance and Economics, Changsha 410205, China

## Abstract

We present a new approach to automatically recognize the pain expression from video sequences, which categorize pain as 4 levels: “no pain,” “slight pain,” “moderate pain,” and “ severe pain.” First of all, facial velocity information, which is used to characterize pain, is determined using optical flow technique. Then visual words based on facial velocity are used to represent pain expression using bag of words. Final pLSA model is used for pain expression recognition, in order to improve the recognition accuracy, the class label information was used for the learning of the pLSA model. Experiments were performed on a pain expression dataset built by ourselves to test and evaluate the proposed method, the experiment results show that the average recognition accuracy is over 92%, which validates its effectiveness.

## 1. Introduction

In recent years, tremendous amounts of researches have been carried out in the field of automatic expressions (such as pain, anger, and sadness) recognition from video sequence. Pain is a subjective and personal experience, and pain recognition is still difficult. There are numerous potential applications for pain recognition. Doctors can recognize pain when patients are experiencing genuine pain so that their pains are taken seriously, like young children who could not self-report pain measures, or many patients in postoperative care or transient states of consciousness, and with severe disorders requiring assisted breathing, among other conditions [1, 2]. Real-time automatic system can be trained which could potentially provide significant advantage in patient care and cost reduction.

Measuring or monitoring pain is normally conducted via self-report as it is convenient and requires no special skill or staffing. However, self-report measures cannot be used when patients cannot communicate verbally. Many researchers have pursued the goal of obtaining a continuous objective measure of pain through analyses of tissue pathology, neurological “signatures,” imaging procedures, testing of muscle strength, and so on [3]. These approaches have been fraught with difficulty because they are often inconsistent with other evidence of pain [3], in addition to being highly invasive and constraining to the patient.

The experience of pain is often represented by changes in facial expression. So, facial expression is considered to be the most reliable source of information when judging the pain intensity experienced by another. In the past several years, significant efforts have been made to identify reliable and valid facial indicators of pain [4–14]. In [4, 5], an approach was developed to automatically recognize acute pain; active appearance models (AAM) were used to decouple shape and appearance parameters from face images; based on AAM, three pain representations were derived. And then SVM were used to classify pain. In [6–10], Prkachin and Solomon validated a facial action coding system (FACS) based measure of pain that can be applied on a frame-by-frame basis. But these methods require manual labeling of facial action units or other observational measurements by highly trained observers [15, 16], which is both timely and costly. Most must be performed offline, which makes them ill-suited for real-time applications in clinical settings. In [11], a robust approach for pain expression recognition was presented using video sequences. An automatic face detector is employed which uses skin color modeling to detect human face in the video sequence. The pain affected portions of the face are obtained by using a mask image. The obtained face images are then projected onto a feature space, defined by Eigenfaces, to produce the biometric template. Pain recognition is performed by projecting a new image onto the feature spaces spanned by the Eigenfaces and then classifying the painful face by comparing its position in the feature spaces with the positions of known individuals. Zhang and Xia [12] used supervised locality preserving projections (SLPP) to extract feature of pain expression, and multiple kernels support vector machines (MKSVM) are employed for recognizing pain expression. Methods described above used static features to character pain expression, but these static features cannot fully represent pain.

In this paper, we propose a method for automatically inferring pain form video sequences. This approach includes two steps: extracting feature of pain expression and classifying pain expression. In the extracting feature, features of pain expression are extracted by motion descriptor based on optical flow. Then we convert facial velocity information to visual words using “bag-of-words” models, and pain expression is represented by a number of visual words; final pLSA model is used for pain expression recognition. In addition, in order to improve the recognition accuracy, the class label information was used for the learning of the pLSA model.

The paper is structured as follows. After reviewing related work in this section, we describe the pain feature extraction based on optical flow technique and “bag-of-words” models in Section 2.  Section 3 gives details of pLSA model for recognizing pain expression.  Section 4 shows experiment result, also comparing our approach with three state-of-the-art methods, and the conclusions are given in the final section.

## 2. Pain Expression Representation

### 2.1. Facial Velocity Feature

According to the physiology, the experience of pain is often represented by changes in facial expression and the expression is a dynamic event; it is must be represented by the motion information of the face. So, we use facial velocity features to characterize pain. The facial velocity features (optical flow vector) are estimated by optical flow model, and each pain expression was coded on a 4-level intensity dimension (A–D): “no pain,” “slight pain,” “moderate pain,” and “severe pain.”

Given a stabilized video sequence in which the face of a person appears in the center of the field of view, we compute the facial velocity (optical flow vector) **u** = (*u*_*x*_, *u*_*y*_) at each frame using optical flow equation, which is expressed as

(1) $$\begin{array}{c} I_{x}u_{x}+I_{y}u_{y}+I_{t}=0, \end{array}$$

where

(2) $$\begin{array}{c} I_{x}=\frac{\partial I}{\partial x},\;I_{y}=\frac{\partial I}{\partial y},\;I_{t}=\frac{\partial I}{\partial t},\\ u_{x}=\frac{dx}{dt},\;u_{y}=\frac{dy}{dt}, \end{array}$$

where (*x*, *y*, *t*) is the image in pixel (*x*, *y*) at time *t*, where *I*(*x*, *y*, *t*) is the intensity at pixel (*x*, *y*) and time *t*, *u*_*x*_, *u*_*y*_ are the horizontal and vertical velocities in pixel (*x*, *y*).

We can obtain **u** = (*u*_*x*_, *u*_*y*_) by minimizing the objective function:

(3) $$\begin{array}{c} C=\int_{D}\left[\lambda^{2}{||\nabla u||}^{2}+{\left(\nabla I\cdot u+I_{t}\right)}^{2}\right]dx\;dy. \end{array}$$

There are many methods to solve the optical flow equation. We use the iterative algorithm [17] to compute the optical flow velocity:

(4) $$\begin{array}{c} u_{x}^{k+1}={\overset{-}{u}}_{x}^{k}-\frac{I_{x}\left[I_{x}{\overset{-}{u}}_{x}^{k}+I_{y}{\overset{-}{u}}_{y}^{k}+I_{t}\right]}{\lambda +I_{x}^{2}+I_{y}^{2}},\\ u_{y}^{k+1}={\overset{-}{u}}_{y}^{k}-\frac{I_{y}\left[I_{x}{\overset{-}{u}}_{x}^{k}+I_{y}{\overset{-}{u}}_{y}^{k}+I_{t}\right]}{\lambda +I_{x}^{2}+I_{y}^{2}}, \end{array}$$

where *k* is the number of iterations, initial value of velocity *u*_*x*_^0^ = *u*_*y*_^0^ = 0, and ${\overset{-}{u}}_{x}^{k},\;{\overset{-}{u}}_{y}^{k}$ is the average velocity of the neighborhood of point (*x*, *y*).

The optical flow vector field **u** is then split into two scalar fields *u*_*x*_ and *u*_*y*_, corresponding to the *x* and *y* components of **u** [18]. *u*_*x*_ and *u*_*y*_ are further half-wave rectified into four nonnegative channels *u*_*x*_^+^,*u*_*x*_^−^,*u*_*y*_^+^, and *u*_*y*_^−^, so that *u*_*x*_ = *u*_*x*_^+^ − *u*_*x*_^−^ and *u*_*x*_ = *u*_*x*_^+^ − *u*_*x*_^−^. These four nonnegative channels are then blurred with a Gaussian kernel and normalized to obtain the final four channels *ub*_*x*^+^_,*ub*_*x*^−^_,*ub*_*x*^−^_, and *ub*_*x*^−^_.

Facial pain expressionisrepresented by velocity features that are composed of the channels *ub*_*x*^+^_,*ub*_*x*^−^_,*ub*_*y*^+^_, and *ub*_*y*^−^_ of all pixels in facial image. Because pain expression can be regarded as facial motion, the velocity features can describe pain effectively, in addition to the velocity features having been shown to perform reliably with noisy image sequences [18], and have been applied in various tasks, such as action classification and motion synthesis. But the dimension of these velocity features is too high (4 × *N* × *N*, where *N* × *N* is image size) to be used directly for recognition and, so, we convert these velocity features into visual words using “bag of words” [19, 20].

### 2.2. Visual Words for Characterizing Pain

The “bag-of-words” model was originally proposed for analyzing text documents, where a document is represented as a histogram over word counts.

In this paper, each facial image is divided into blocks whose size is *L* × *L*, and each image block is represented by optical flow vector of all pixels in the block. On this basis, pain is represented by visual words using the method of BoW (bag of words).

To construct the codebook, we randomly select a subset from all image blocks; then, we use *k*-means clustering algorithms to obtain *V* clusters. Codewords are then defined as the centers of the obtained clusters, namely, visual words. In the end, each face image is converted to the “bag-of-words” representation by appearance times of each codeword in the image that is used to represent the image, namely, BoW histogram.

The step for characterizing pain is as follows.

**Step 1**

Optical flow channels *ub*_*x*^+^_,*ub*_*x*^−^_,*ub*_*y*^+^_, and *ub*_*y*^−^_ are computed.

**Step 2**

Each facial image is divided into *n* × *n* blocks, which is represented by optical flow vector of all pixels in the block.

**Step 3**

Vision words are obtained using *k*-means clustering algorithms.

**Step 4**

Pain expression is represented by BoW histogram *d*:

(5) $$\begin{array}{c} d=\left\{n\left(I,w_{1}\right),\ldots ,n\left(I,w_{j}\right),\ldots ,n\left(I,w_{M}\right)\right\}, \end{array}$$

where *n*(*I*, *w*_*j*_) is the number of visual word *w*_*j*_ included in image and *M* is the number of vision words in word sets.

**Figure 1:**
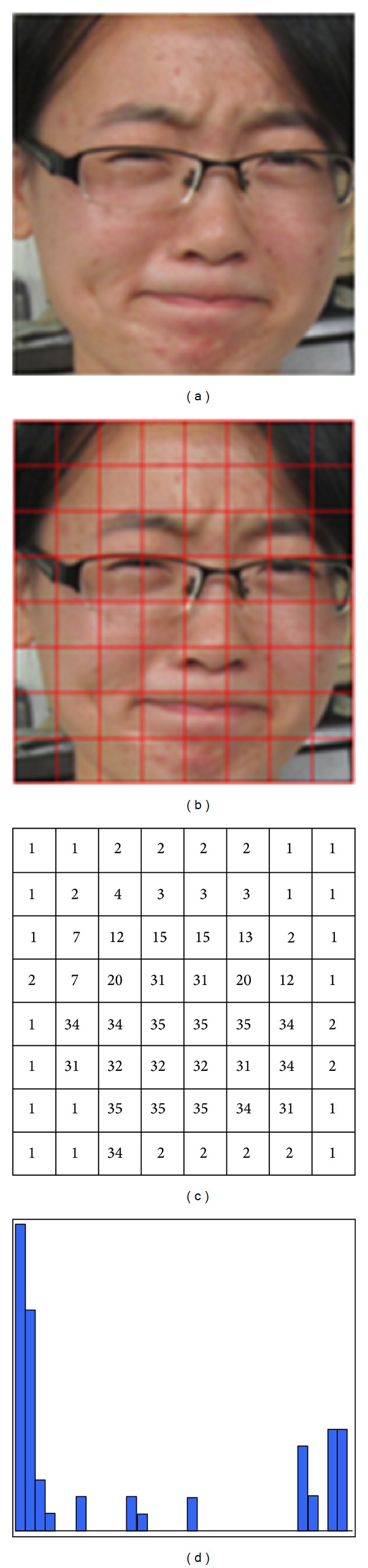
The processing pipeline of the “bag-of-words” representation: (a) give an image, (b) divide into *L* × *L* blocks, (c) represent each block by a “visual word,” and (d) ignore the ordering of words and represent the facial image as a histogram over “visual words.”

Figure 1 shows an example of our “bag-of-words” representation.

## 3. pLSA-Based Pain Expression Recognition

We use the pLSA models [21] to learn and recognize human pain. Our approach is directly inspired by a body of work on using generative topic models for visual recognition based on the “bag-of-words” paradigm. The pLSA models have been applied to various computer vision applications, such as scene recognition, object recognition, action recognition, and human detection [22–26].

### 3.1. Probabilistic Latent Semantic Analysis (pLSA)

**Figure 2:**
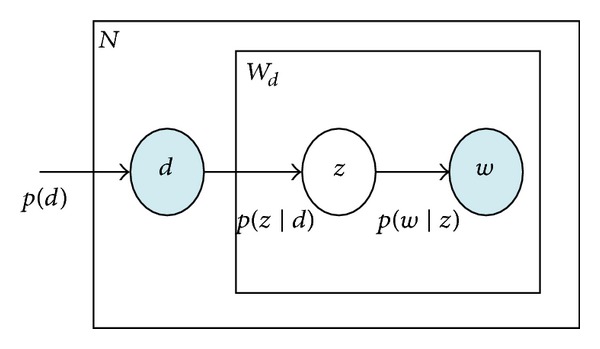
Graph model of pLSA. Nodes represent random variables. Shaded nodes are observed variables and unshaded ones are unseen variables. The plates stand for repetitions.

pLSA is a statistical generative model that associates documents and words via the latent topic variables, which represents each document as a mixture of topics. We briefly outline the principle of the pLSA in this subsection. The model of pLSA is shown in Figure 2.

Suppose document, word, and topic are represented by *d*_*i*_, *w*_*j*_, and* z*_*k*_, respectively. The joint probability of document *d*_*i*_, topic* z*_*k*_, and word *w*_*j*_ can be expressed as

(6) $$\begin{array}{c} p\left(d_{i},z_{k},\omega_{j}\right)=p\left(\omega_{j}\;|\;z_{k}\right)p\left(z_{k}\;|\;d_{i}\right)p\left(d_{i}\right), \end{array}$$

where *p*(*ω*_*j*_ | *z*_*k*_) is the probability of word *ω*_*j*_ occurring in pain category *z*_*k*_, *p*(*z*_*k*_ | *d*_*i*_) is the probability of topic *z*_*k*_ occurring in image *d*_*i*_, and *p*(*d*_*i*_) can be considered as the prior probability of *d*_*i*_. The conditional probability of *p*(*ω*_*j*_ | *d*_*i*_) can be obtained by marginalizing over all the topic variables *z*_*k*_:

(7) $$\begin{array}{c} p\left(\omega_{j}\;|\;d_{i}\right)=\underset{k}{\sum }p\left(z_{k}\;|\;d_{i}\right)p\left(\omega_{j}\;|\;z_{k}\right). \end{array}$$

Denote *n*(*d*_*i*_, *ω*_*j*_) as the occurrence of word *ω*_*j*_ in image *d*_*i*_; the prior probability *p*(*d*_*i*_) can be modeled as

(8) $$\begin{array}{c} p\left(d_{i}\right)\propto \sum_{j}n\left(d_{i},w_{j}\right). \end{array}$$

A maximum likelihood estimation of *p*(*ω*_*j*_ | *z*_*k*_) and *p*(*z*_*k*_ | *d*_*i*_) is obtained by maximizing the function using the expectation maximization (EM) algorithm. The objective likelihood function of the EM algorithm is

(9) $$\begin{array}{c} l=\underset{i}{\prod }\underset{j}{\prod }p{\left(\omega_{j}\;|\;d_{i}\right)}^{n(w_{j},d_{i})} \end{array}$$

or

(10) $$\begin{array}{c} l=\underset{i}{\sum }\underset{j}{\sum }n\left(d_{i},w_{j}\right)\log p\left(\omega_{j}\;|\;d_{i}\right). \end{array}$$

The EM algorithm consists of two steps: an expectation (E) step computes the posterior probability of the latent variables, and a maximization (M) step maximizes the completed data likelihood computed based on the posterior probabilities obtained from E-step. Both steps of the EM algorithm for pLSA parameter estimate are listed below. 

*E-Step*. Given *p*(*ω*_*j*_ | *z*_*k*_) and *p*(*z*_*k*_ | *d*_*i*_), estimate *p*(*z*_*k*_ | *d*_*i*_, *w*_*j*_):

(11) $$\begin{array}{c} p\left(z_{k}\;|\;d_{i},w_{j}\right)=\frac{p\left(\omega_{j}\;|\;z_{k}\right)p\left(z_{k}\;|\;d_{i}\right)}{\sum_{l}^{}p\left(w_{j}\;|\;z_{l}\right)p\left(z_{l}\;|\;d_{i}\right)}. \end{array}$$

*M-Step*. Given the estimated *p*(*z*_*k*_ | *d*_*i*_, *w*_*j*_) in E-Step and *n*(*d*_*i*_, *w*_*j*_), estimate *p*(*w*_*j*_ | *z*_*k*_) and *p*(*z*_*k*_ | *d*_*i*_):

(12) $$\begin{array}{c} p\left(\omega_{j}\;|\;z_{k}\right)=\frac{\sum_{i}^{}n\left(d_{i},w_{j}\right)p\left(z_{k}\;|\;d_{i},w_{j}\right)}{\sum_{i}^{}\sum_{h}^{}n\left(d_{i},w_{h}\right)p\left(z_{k}\;|\;d_{i},w_{h}\right)}, \end{array}$$

(13) $$\begin{array}{c} p\left(z_{k}\;|\;d_{i}\right)=\frac{\sum_{j}^{}n\left(d_{i},w_{j}\right)p\left(z_{k}\;|\;d_{i},w_{j}\right)}{n\left(d_{i}\right)}, \end{array}$$

where *n*(*d*_*i*_) = ∑_*j*_ *n*(*d*_*i*_, *w*_*j*_) is the length of document *d*_*i*_.

Given a new document, the conditional probability distribution over aspect *p*(*z* | *d*_new_) can be inferred by maximizing the likelihood of *d*_new_ using a fixed word-aspect distribution *p*(*ω*_*j*_ | *z*_*k*_) learned from the observed data [21]. The iteration of inferring *p*(*z* | *d*_new_) is the same as the learning process except that the word-topic distribution *p*(*ω*_*j*_ | *z*_*k*_) in (12) is a fixed value, that is, learned from training data.

### 3.2. pLSA-Based Pain Expression Recognition

In this paper, we treat each block in an image as a single word *w*_*j*_, an image as a document *d*_*i*_, and a pain category as a topic variable* z*_*k*_. For the task of pain classification, our goal is to classify a new face image to a specific pain class. During the inference stage, given a testing face image and the document specific coefficients *p*(*z*_*k*_ | *d*_test_), we can treat each aspect in the pLSA model as one class of pains. So, the pain categorization is determined by the aspect corresponding to the highest *p*(*z*_*k*_ | *d*_test_). The pain category *k* of *d*_test_ is determined as

(14) $$\begin{array}{c} k=\arg\;\underset{k}{\max}p\left(z_{k}\;|\;d_{\text{test}}\right). \end{array}$$

**Figure 3:**
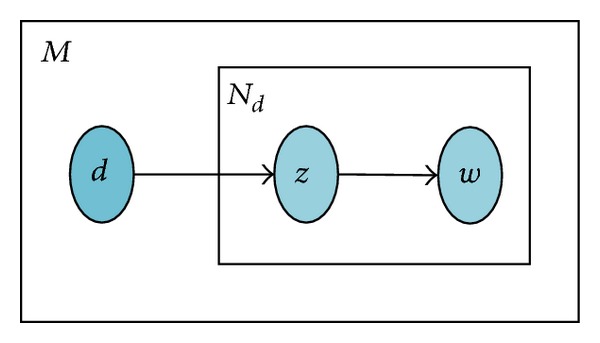
Graph model of SpLSA. Nodes represent random variables. Shaded nodes are observed variables and unshaded ones are unseen variables. The plates stand for repetitions.

For pain recognition with large amount of training data, this would result in long training time. In this paper, we adopt a supervised algorithm to train pLSA, which is similar to [27]. Each image has class label information in the training images, which is important for the classification task. Here, we make use of this class label information in the training images for the learning of the pLSA model, since each image directly corresponds to a certain pain class on train sets; the image for training data becomes observable. This model is called supervised pLSA (SpLSA). The graphical model of SpLSA is shown in Figure 3.

The parameter *p*(*w*_*j*_ | *z*_*k*_) in the training step defines the probability of a word *w*_*j*_ drawing from a topic* z*_*k*_. Letting each topic in pLSA correspond to a pain category, the distribution *p*(*w*_*j*_ | *z*_*k*_) in the training can be simply estimated as

(15) $$\begin{array}{c} p\left(\omega_{j}\;|\;z_{k}\right)=\frac{n_{j,k}}{n_{k}}, \end{array}$$

where *n*_*k*_ is the number of the images corresponding to the *k*th pain class and *n*_*j*,*k*_ is the number of the *j*th word (block) in the images corresponding to the *k*th pain class. This means that the *p*(*ω*_*j*_ | *z*_*k*_) calculated by this way can be used to initialize the *p*(*w* | *z*) in the EM algorithm for model learning, which makes the EM algorithm converge more quickly. The supervised training of pLSA is summarized in Algorithm 1. Once the distribution *p*(*w* | *z*) is computed by the EM algorithm, for a testing face image *d*_test_, the posterior distribution *p*(*z*_*k*_ | *d*_test_) can be calculated the same as in original pLSA. The training of pLSA for classification is summarized in Algorithm 2.

**Algorithm 1**

Supervised training of the pLSA.

**Step 1**

For all *k* and *j*, calculate

(16) $$\begin{array}{c} p\left(\omega_{j}\;|\;z_{k}\right)=\frac{n_{j,k}}{n_{k}}, \end{array}$$

as the initialization of the *p*(*w* | *z*) and random initialization of the *p*(*z* | *d*).

**Step 2**

E-Step: for all (*d*_*i*_, *w*_*j*_) pairs, calculate

(17) $$\begin{array}{c} p\left(z_{k}\;|\;d_{i},w_{j}\right)=\frac{p\left(\omega_{j}\;|\;z_{k}\right)p\left(z_{k}\;|\;d_{i}\right)}{\sum_{l}p\left(w_{j}\;|\;z_{l}\right)p\left(z_{l}\;|\;d_{i}\right)}. \end{array}$$

**Step 3**

M-Step: substitute *p*(*z*_*k*_ | *d*_*i*_, *w*_*j*_) as calculated in Step 2; for all *k* and  *j*, calculate

(18) $$\begin{array}{c} p\left(\omega_{j}\;|\;z_{k}\right)=\frac{\sum_{i}n\left(d_{i},w_{j}\right)p\left(z_{k}\;|\;d_{i},w_{j}\right)}{\sum_{m}\sum_{i}n\left(d_{i},w_{m}\right)p\left(z_{k}\;|\;d_{i},w_{m}\right)}. \end{array}$$

**Step 4**

M-Step: substitute *p*(*z*_*k*_ | *d*_*i*_, *w*_*j*_) as calculated in Step 2; for all *i* and *k*, calculate

(19) $$\begin{array}{c} p\left(z_{k}\;|\;d_{i}\right)=\frac{\sum_{j}n\left(d_{i},w_{j}\right)p\left(z_{k}\;|\;d_{i},w_{j}\right)}{n\left(d_{i}\right)}. \end{array}$$

**Step 5**

Repeat Steps 2–4 until the convergence condition is met.

The supervised training algorithm not only makes the training more efficient, but also improves the overall recognition accuracy significantly.

**Algorithm 2**

Training of the pLSA for classification

**Step 1**

For all *k* and *j*, calculate

(20) $$\begin{array}{c} p\left(\omega_{j}\;|\;z_{k}\right)=\frac{n_{j,k}}{n_{k}}. \end{array}$$

**Step 2**

E-Step: for all (*d*_test_, *w*_*j*_) pairs, calculate

(21) $$\begin{array}{c} p\left(z_{k}\;|\;d_{\text{test}},w_{j}\right)=\frac{p\left(\omega_{j}\;|\;z_{k}\right)p\left(z_{k}\;|\;d_{\text{test}}\right)}{\sum_{l}p\left(w_{j}\;|\;z_{l}\right)p\left(z_{l}\;|\;d_{\text{test}}\right)}. \end{array}$$

**Step 3**

Partial M-Step: fix *p*(*w*_*j*_ | *z*_*k*_) as calculated in Step 1; for all *k*, calculate

(22) $$\begin{array}{c} p\left(z_{k}\;|\;d_{\text{test}}\right)=\frac{\sum_{j}n\left(d_{\text{test}},w_{j}\right)p\left(z_{k}\;|\;d_{\text{test}},w_{j}\right)}{n\left(d_{\text{test}}\right)} \end{array}$$

**Step 4**

Repeat Steps 2 and 3 until the convergence condition is met.

**Step 5**

Calculate pain class

(23) $$\begin{array}{c} k=\arg\;\underset{k}{\max}p\left(z_{k}\;|\;d_{\text{test}}\right). \end{array}$$

## 4. Experimental Results and Analysis

The effectiveness of the proposed algorithm was verified by using C++ and MATLAB hybrid implementation on a PC with Pentium 3.2 GHz processor and 4 G RAM.

**Figure 4:**
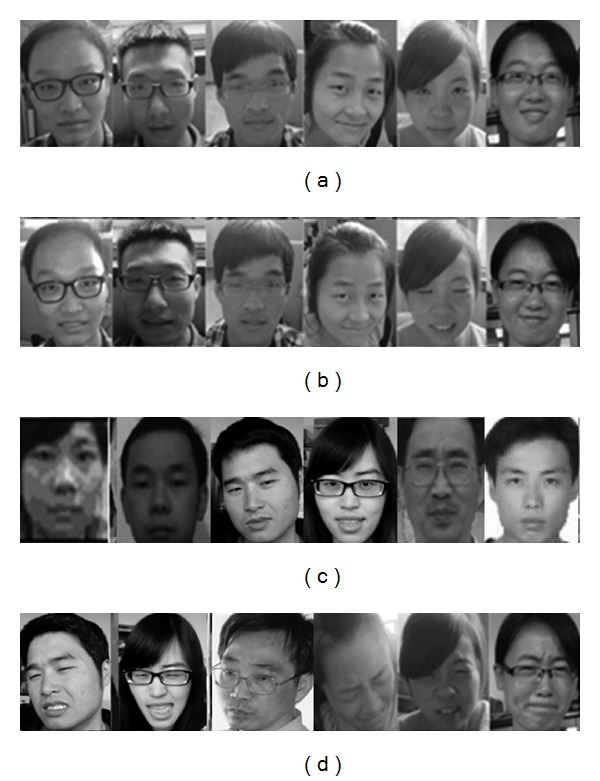
Examples of recognizing pain expression from facial videos. (a) No pain, (b) slight pain, (c) moderate pain, and (d) severe pain.

We have built a database of painful and normal face images. In this database, there are four groups of images (“no pain,” “slight pain,” “moderate pain,” and “severe pain”), and each group includes 20 males and 20 females. The face images were taken under various laboratory-controlled lighting conditions, and each face image was normalized to a size of 64 × 64. Some sample images are shown in Figure 4.

In experiments, 30 face images per class are randomly chosen for training, while the remaining images are used for testing. We preprocessed these images by aligning and scaling them so that the distances between the eyes were the same for all images and also ensuring that the eyes occurred in the same coordinates of the image. We run the system 5 times and obtain 5 different training and testing sample sets. The recognition rates were found by averaging the recognition rate of each run.

**Figure 5:**
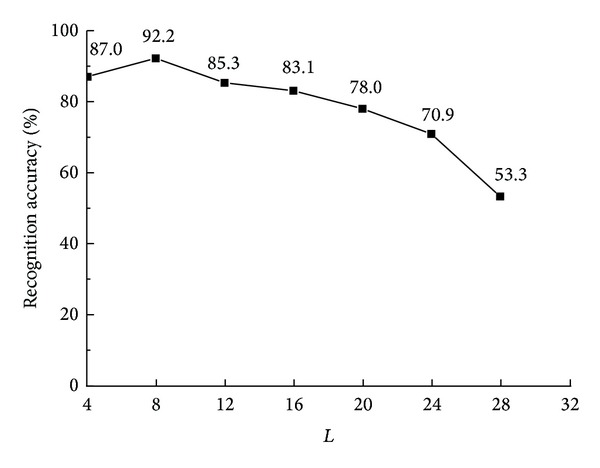
Recognition accuracy curve with different block sizes.

Each facial image is divided into blocks whose size is *L* × *L*. First, we studied the effect of the size of image block on the recognition accuracy.  Figure 5 represents the recognition accuracy curve with different block sizes *L*. It can be concluded that the accuracy peaks when the block sizes *L* is 8. Therefore *L* is set as 8.

**Figure 6:**
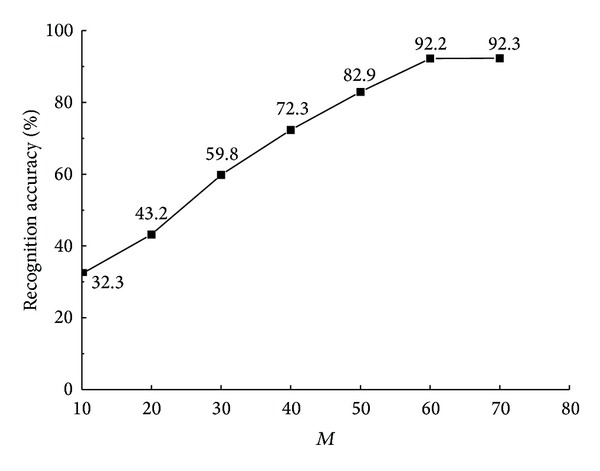
Relation curve between *M* and accuracy.

In order to determine the value of *M*, that is, the number of the visual word set, the relation between *M* and recognition accuracy was observed, which is displayed in Figure 6. It is revealed in Figure 4 that the recognition accuracy is risen up at the beginning with the increasing of *M* recognition and if *M* is larger than or equal to 60, the recognition accuracy is stabled to 0.922. As a result, *M* is set as 60.

To examine the accuracy of our proposed pain recognition approach, we compare our method to three state-of-the-art approaches for pain recognition using the same data. The first method is “AAM + SVM” [4], which used active appearance models (AAM) to extract face features and SVM to classify pain. The second method is “Eigenimage” [11], which used Eigenface for pain recognition. The third method is “SLPP + MKSVM” [12], which used SLPP to extract feature of pain expression and multiple kernels support vector machines (MKSVM) for recognizing. 200 different expression images are used for this experiment. Some images contain the same person but in different moods. The recognition results are presented in the confusion matrices shown in Table 1. Each cell in the confusion matrix is the average results; our results are at the upper left; the results of “AAM + SVM,” “Eigenimage,” and “SLPP + MKSVM” are presented in the upper right, the lower left, and the lower right, respectively. where A, B, C, and D indicate no pain, slight pain, moderate pain, and severe pain, respectively. As Table 1 shows, our method improves the recognition accuracies in all categories. It achieves 92.2% average recognition rate, whereas “AAM + SVM” obtain 81.2%, “Eigenimage” gets 82.5%, and “SLPP + MKSVM” attains 86.5%, as shown in Table 2. The reason is that we improve the recognition accuracy in the two stages of pain feature extraction and pain expression recognition. In the stage of pain feature extraction, we use motion features that are reliable with noisy image sequences and describe pain effectively, while other methods used static features, which cannot effectively describe the expression of pain. In the stage of expression recognition, we use bag-of-words framework and pLAS model to classify expression images. In addition, we make use of this class label information in the training images for the learning of the pLSA model, which can improve the overall recognition accuracy significantly.

## 5. Conclusion

Pain recognition can provide significant advantage in patient care and cost reduction. In this paper, we present a novel method to recognize the pain expression and give the pain level at the same time. The main contribution can be concluded as follows.

1. Visual words are used for pain expression. Optical flow model is used for extracting facial velocity features; then we convert facial velocity features into visual words using “bag-of-words” models.
2. We use pLSA topic models for pain expression recognition. In our models the “latent topics” directly correspond to different pain expression categories. In addition, in order to improve the recognition accuracy, the class label information was used for the learning of the pLSA model.
3. Experiments were performed on a pain expression dataset built by ourselves and evaluate the proposed method. Experimental results reveal that the proposed method performs better than previous ones.

## Figures and Tables

**Table:** (a)

	A	B	C	D
A	**0.95 **	0.04	0.01	0.00
B	0.04	**0.91 **	0.05	0.00
C	0.01	0.03	**0.91 **	0.05
D	0.00	0.02	0.05	**0.93 **

**Table:** (b)

	A	B	C	D
A	**0.84 **	0.10	0.03	0.02
B	0.11	**0.78 **	0.08	0.03
C	0.03	0.09	**0.79 **	0.08
D	0.01	0.05	0.12	**0.83 **

**Table:** (c)

	A	B	C	D
A	**0.85 **	0.11	0.03	0.01
B	0.10	**0.80 **	0.08	0.02
C	0.02	0.08	**0.79 **	0.08
D	0.01	0.04	0.12	**0.84 **

**Table:** (d)

	A	B	C	D
A	**0.88 **	0.09	0.02	0.01
B	0.09	**0.82 **	0.07	0.02
C	0.02	0.08	**0.83 **	0.07
D	0.00	0.02	0.08	**0.90 **

**Table 2:** Comparison of different reported results on pain dataset.

Method	Accuracy (%)
Our method	92.20
“AAM + SVM” [4]	81.20
“Eigenimage” [11]	82.50
“SLPP + MKSVM” [13]	86.50
